# Supplementary material for: The Effects of DNA Methylation on Cytoplasmic Male Sterility in Sugar Beet
Source: Int J Mol Sci. 2024 Jan 17;25(2):1118. doi: 10.3390/ijms25021118 (PMC10817047; doi:10.3390/ijms25021118)

Figure S1 Methylation level and gene expression level of DMGs between CMS line and maintainer line

A: Relative expression levels of differentially methylated genes, B: methylation levels of differentially methylated genes.

Note: "*" as the p value in 0.01-0.05, "**" p value in 0.001-0.01 "***" p values < 0.001

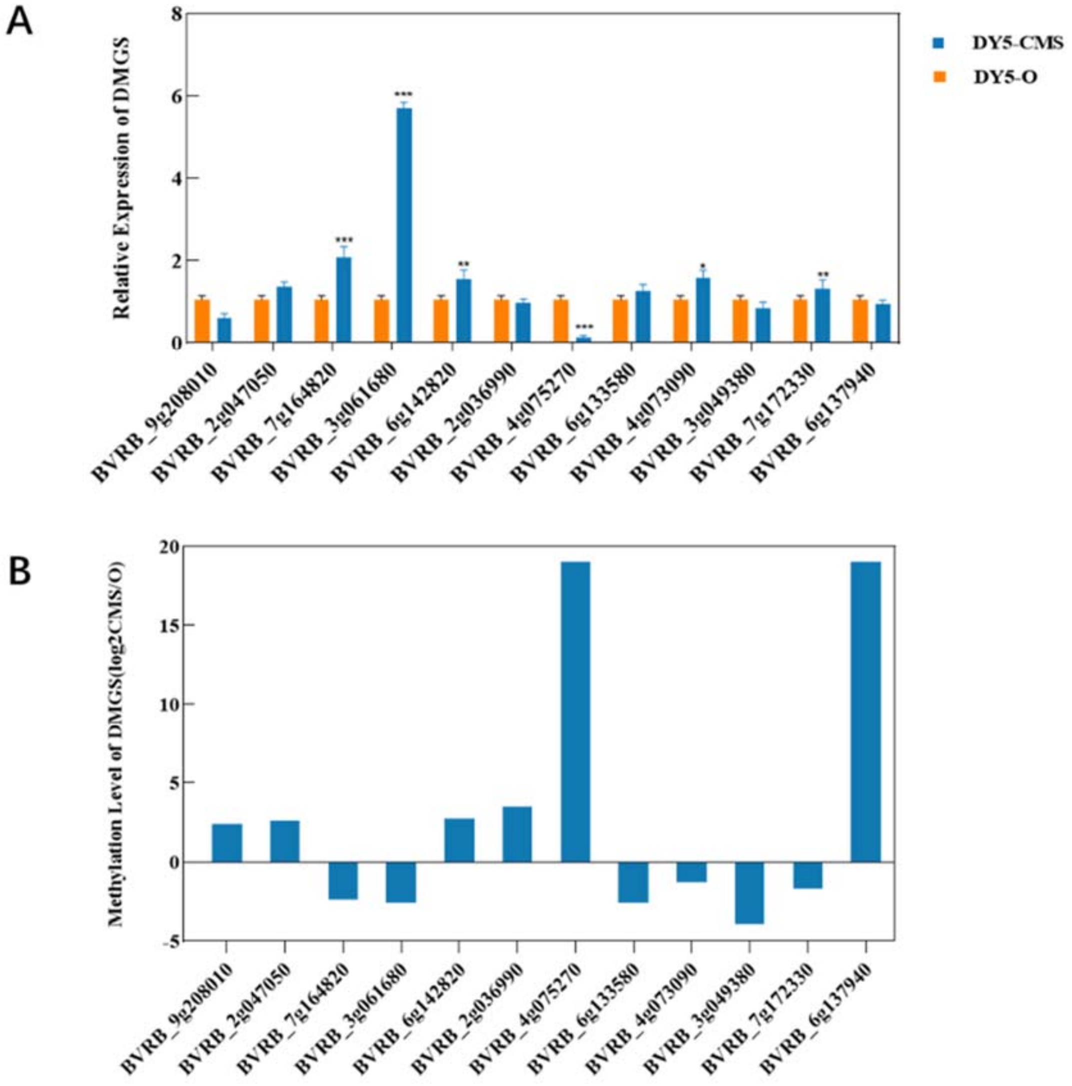

Supplement: Supplementary file 1 [file ijms-25-01118-s001.zip › Figure S1.pdf]
